# Genome-Wide Screening and Characterization of the *Dof* Gene Family in Physic Nut (*Jatropha curcas* L.)

**DOI:** 10.3390/ijms19061598

**Published:** 2018-05-29

**Authors:** Peipei Wang, Jing Li, Xiaoyang Gao, Di Zhang, Anlin Li, Changning Liu

**Affiliations:** 1 Key Laboratory of Tropical Plant Resource and Sustainable Use, Xishuangbanna Tropical Botanical Garden, Chinese Academy of Sciences, Kunming 650223, China; wangpeipei@xtbg.ac.cn (P.W.); lijing3@xtbg.ac.cn (J.L.); gaoxiaoyang@xtbg.ac.cn (X.G.); zhangdi_net@foxmail.com (D.Z.); lianlin@xtbg.ac.cn (A.L.); 2 Faculty of Life Sciences, University of Chinese Academy of Sciences, Beijing 100049, China

**Keywords:** *Jatropha curcas*, *Dof* gene family, transcription factor, phylogenetic analysis, gene expression analysis

## Abstract

Physic nut (*Jatropha curcas* L.) is a species of flowering plant with great potential for biofuel production and as an emerging model organism for functional genomic analysis, particularly in the Euphorbiaceae family. DNA binding with one finger (Dof) transcription factors play critical roles in numerous biological processes in plants. Nevertheless, the knowledge about members, and the evolutionary and functional characteristics of the *Dof* gene family in physic nut is insufficient. Therefore, we performed a genome-wide screening and characterization of the *Dof* gene family within the physic nut draft genome. In total, 24 *JcDof* genes (encoding 33 JcDof proteins) were identified. All the *JcDof* genes were divided into three major groups based on phylogenetic inference, which was further validated by the subsequent gene structure and motif analysis. Genome comparison revealed that segmental duplication may have played crucial roles in the expansion of the *JcDof* gene family, and gene expansion was mainly subjected to positive selection. The expression profile demonstrated the broad involvement of *JcDof* genes in response to various abiotic stresses, hormonal treatments and functional divergence. This study provides valuable information for better understanding the evolution of *JcDof* genes, and lays a foundation for future functional exploration of *JcDof* genes.

## 1. Introduction

Physic nut (*Jatropha curcas* L.) is a perennial small tree in the spurge family, Euphorbiaceae, with a high seed-oil content (40–50%). It can grow easily in barren soil and endure drought and saline environments, thus, having a broad adaptability in various agro-climatic conditions. Given its great potential for biofuel production, nowadays, Physic nut is attracting much attention due to the gradual depletion and a cost increase of fossil energy resources [1,2]. However, there are still a number of challenges in physic nut industries. For example, most physic nut germplasms are monoecious with very low ratios of female to male flowers (approximately female:male = 1:13–29), which considerably reduces the seed yield in physic nut [3,4]. Another serious drawback to the use of physic nut is the presence of toxic components, such as lectin, trypsin inhibitor, and phorbol esters, in all parts of the plant [5]. Therefore, in-depth understanding of the structure and function of key gene families and metabolic pathways of physic nut is essential for improving its crop productivity and commercialization.

Additionally, physic nut is a potential model organism for functional genomic analysis, particularly in Euphorbiaceae. Physic nut is a diploid species (2*n* = 22) [6], with a relatively small genome size (approximately 416Mbp) compared to other members of the Euphorbiaceae [7,8]. According to the most recently updated version, the assembled genome has a total length of 320.5 Mbp consisting of 27,127 putative protein-coding genes, and most of the scaffolds have been anchored on the genetic linkage map [9]. In addition, high-throughput sequencing has mushroomed over the past decade, stimulating the transcriptome profiling analyses. Gene expression profiles of physic nut from different tissues, developmental stages, and biotic/abiotic stresses were constructed [10,11]. Additionally, more than 170 biosamples of physic nut are publicly available from NCBI (National Coalition Building Institute) (as of 5 February 2018). All these genomic and transcriptional data provided a valuable resource for basic and applied studies in physic nut.

Transcription factors (TFs), also known as trans-acting elements, are DNA-binding proteins that specifically bind cis-acting elements in the eukaryotic promoters to activate or inhibit transcriptional regulation [12,13]. As its important roles in the regulation of plant gene expression [14], the structure and function of TFs are now becoming research hotspots in plant molecular biology. The gene expression involves different classes of TFs, which have evolved to regulate a variety of plant-specific genes or signals [15,16]. The *Dof* gene family is a typical example of such TFs. The Dof proteins typically have 200–400 amino acids and contain two main functional domains. One is the Dof domains in the N-terminal domain includes a highly-conserved single zinc-finger structure formed by a CX2CX21CX2C motif, where one Zn^2+^ can covalently combine with four Cys residues. The other one is a regulatory *C*-terminal domain [17,18,19]. For instance, the transcription activation domain of the maize *ZmDof1* gene is formed by 44 amino acid residues located at the C-terminal domain [20]. In spite of high level homology in the Dof domain, the rest of the amino acid sequences in the proteins are divergent, coinciding with their expected diverse functions [19].

Previous studies disclosed a variety of roles of Dof proteins in gene expression regulation when associated with plant-specific phenomena, including metabolism, photoperiodic regulation, phytohormone responses, defense responses, and other aspects of plant development [21,22,23]. Maize *Dof1* (*ZmDof1*) is the first member of the *Dof* gene family identified in plants which may be involved in novel molecular mechanisms underlying tissue-specific and light-regulated gene expression in plants [24]. In addition, recent studies revealed that two closely-related *Arabidopsis thaliana* L. *Dof* genes, *AtDof3.7* (*DAG1*) and *AtDof2.5* (*DAG2*), are maternal genes involved in the control of seed germination, although their actions are opposite [25]. A rice Dof protein, OsDof3, affected the DNA binding of GAMYB to GARE, which is important for combinational regulation of the transcriptional response to Gibberellic acid (GA), and might be a mediator for GA signaling during germination [19,26]. Although none of roles has been confirmed conclusively, Dof proteins apparently participate in the regulation of various processes.

Until now, the *Dof* genes have been identified and characterized in different plant species, such as *A. thaliana*, *Oryza sativa* L. [27], *Glycine max* L. [28], *Triticuma estivum* L. [29], and *Ricinus communis* L. [30]. However, understanding of the *Dof* gene family members and their evolutionary and functional characteristics in physic nut is limited. Based on the genomic and transcriptional data, we focused on the identification, characterization and functional exploration of the *Dof* genes in physic nut. In total, 33 JcDof proteins (encoded by 24 *JcDof* genes) were identified and characterized. These *JcDof* genes were further divided into three major groups by comparison to their orthologs/paralogs in castor bean (*Ricinus communis* L.) and *A. thaliana*. The gene structure, motif, and phylogenetic analysis revealed that genes within each group exhibited similar gene structure and protein motif arrangements. Segmental duplication probably played crucial roles in the expansion of the *JcDof* gene family, and the gene expansion was mainly subjected to positive selection. The expression profile demonstrated the broad involvement of *JcDof* genes in response to various abiotic stresses and hormonal treatments and their possible functional divergence. Taken together, our results provide valuable information for understanding the *JcDof* genes’ evolution, and lay a foundation for future functional analysis of the *JcDof* genes.

## 2. Results

### 2.1. Identification and Characterization of Dof Genes in Physic Nut

To extensively identify all the Dof candidate members in the physic nut genome, we used a whole-genome scanning to identify genes that encode proteins containing the Dof DNA-binding domain by both BLASTP and HMM profile search. Initially, the Dof protein sequences from *Arabidopsis thaliana* and their HMM profiles of the Dof domain were used as the BLASTP and HMMER query sequences to screen the physic nut genome. Subsequently, it was examined for the presence of the Dof domain using the SMART software and NCBI Conserved Domain database for all the Dof candidate sequences. Eventually, we identified 24 candidates of *Dof* genes in total, represented by 33 transcripts in physic nut (Table S1). Based on their gene loci, we designated each Dof protein uniquely as JcDof-1, and JcDof-2 to JcDof-24.

In addition, we systematically evaluated the basic properties of JcDof protein, including domain position, protein length, molecular weight (Mw), isoelectric point (pI), instability coefficient, and orthologous genes (Table 1). The average length of these Dof protein sequences was 339 amino acid residues and the length mainly centered on the range of 160–518 amino acid residues. Correspondingly, the molecular weights were mainly distributed from 18.2 kDa (JcDof-1) to 55.7 kDa (JcDof-6). The predicted isoelectric point of Dof proteins varied from 4.65 (JcDof-21) to 9.42 (JcDof-3). The instability coefficient of JcDof protein showed a variation from 39.4 (JcDof-17) to 61.74 (JcDof-7.3-5). The location of JcDof protein conserved domain was analyzed by SMART. It was found that the domain positions of JcDof proteins encoded by the same gene (i.e., JcDof proteins that are generated by alternative splicing of the same gene model) were similar, but quite different for those encoded by different genes.

### 2.2. DNA-Binding Domain Conservation Analysis of JcDof Protein

**Figure 1:**
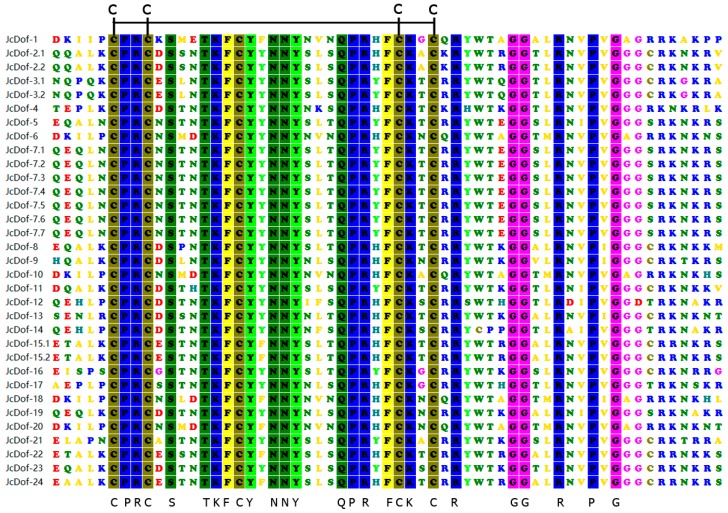
Multiple protein sequences alignments against Dof DNA-binding domain in *JcDof* genes. The identical amino acids are shown in bottom and the four cysteine residues are indicated on top.

Dof protein usually has a DNA-binding domain of approximate 40–60 amino acid residues in the N-terminus. This domain contains a highly-conserved CX2CX21CX2C single zinc-finger structure, which is essential for the zinc finger configuration and loop stability. In this study, the conservation of DNA-binding domain of JcDof proteins was analyzed. Multiple protein sequence alignments against *Dof* DNA-binding domain of JcDof proteins revealed that all of them were highly conserved. Especially, we found 20 highly-conserved (100% identical in all 33 JcDof proteins) amino acids CPRC-S–TKFCY-NNY—QPR-FCK-C in the 29 amino acid-long region which corresponded to the CX2CX21CX2C single zinc-finger structure (Figure 1).

### 2.3. Phylogenetic Analysis and Classification of JcDof Proteins

**Figure 2:**
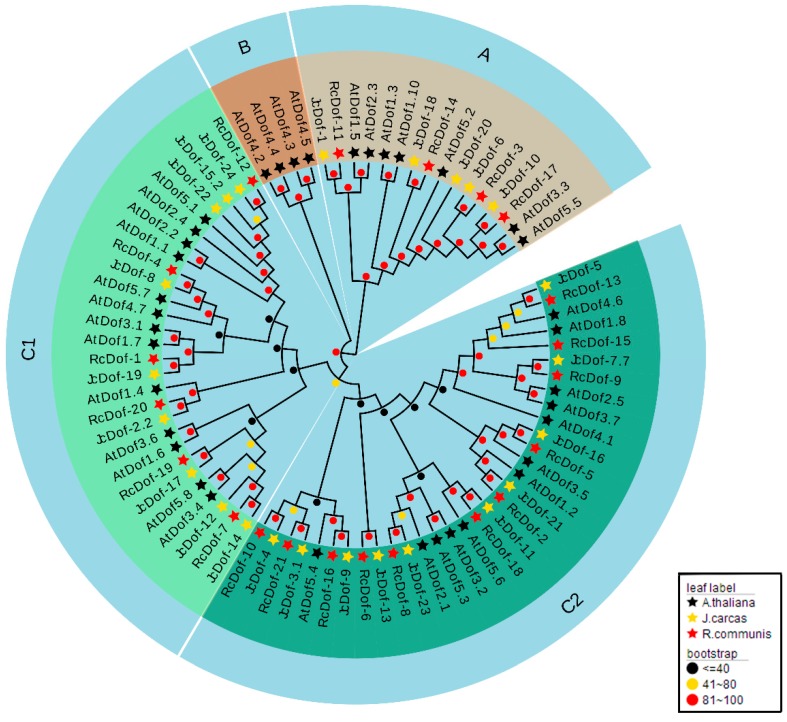
Phylogenetic relationships among *J. curcas*, *A. thaliana*, and *R. communis* Dof proteins. The neighbor-joining tree was created using the MEGA6.0 program (bootstrap value set at 1000). Thirty-six (36) AtDof proteins marked with black pentacle, 24 JcDof proteins marked with yellow pentacle, and 21RcDof proteins marked with red pentacle. The resulting phylogenetic tree was clustered into three major groups (A, B, and C), which were supported by a bootstrap value over 80%. The Dof proteins in Group C were further divided into two sub-groups, C1 and C2, supported by a bootstrap value over 40%. The detailed information of all the Dof proteins is listed in Supplementary Table S2.

To explore the phylogenetic relationships of JcDof proteins, we carried out phylogenetic analysis on Dof proteins from physic nut and other two plant species, including *Ricinus communis*, also from the Euphorbiaceae family, and *A. thaliana*, as an outgroup (detailed information on all of the Dof proteins is listed in Supplementary Table S2). A phylogenetic tree was reconstructed including 24 physic nut, 21 *R. communis* and 36 *A. thaliana* Dof proteins (Figure 2). For each gene, we chose the longest protein formed by alternative splicing. The resulting phylogenetic tree was clustered into three major groups (A, B, and C), and they were considered to be evidentfor distinct phylogenetic lineages, which were supported by a bootstrap value over 80%. The two external nodes at the end of the same clades of phylogenetic tree were likely to represent the closest homologous gene pairs.

Of the three major groups, Group C was the first main clade, containing 19 physic nut Dof proteins, 17 *R. communis* Dof proteins, and 25 *A. thaliana* Dof proteins, which were further divided into two sub-groups, C1 and C2, supported by a bootstrap value over 40%. Group A was the second major clade with five physic nut Dof proteins, four *R. Communis* Dof proteins, and seven *A. Thaliana* Dof proteins. Group B was the minimal clade, with only four proteins. Distinguishingly, the Group B Dof proteins were only found in *Arabidopsis*, which could be explained by species/lineage-specific gene gain or loss events. We further checked the GO (Gene Ontology) annotations of these four *Arabidopsis Dof* genes, and found that comparing with the *Arabidopsis Dof* genes in other groups, two of these four genes (*At4g21030*, *At4g21050*) have some specific annotations, such as “cotyledon development”, “mucilage metabolic process involved in seed coat development”, “regulation of secondary shoot formation”, and “fruit development”, which implied the possible function divergence of Dof genes in group B (Supplementary Table S3 for detailed information). The phylogenetic tree showed that Dofs in the Group A and C were duplicated several times before the divergence of these three species, and were highly conserved among *J. curcas*, *R. communis*, and *A. thaliana*. In addition, the physic nut Dof proteins were more closely related, evolutionarily, to *R. communis* than to the *Arabidopsis* Dof proteins.

### 2.4. JcDof Gene Structures and Conserved Motifs in JcDof Proteins

**Figure 3:**
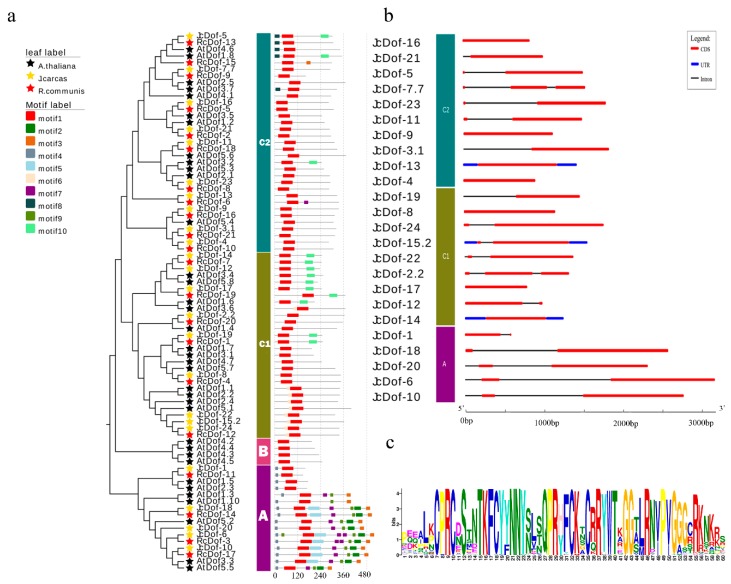
*JcDof* gene structures and conserved motifs in JcDof proteins. (**a**) The distribution of 10 conserved motifs in Dof proteins; (**b**) Gene structures of *JcDof* genes. CDS, UTR and introns were depicted by filled red boxes, blue boxes, and single black lines; and (**c**) Motif-1, corresponding to theCX2CX21CX2C single zinc-finger structure. The detailed motif’s sequences are shown in Figure S1 and Table S4.

Introns and exons are the backbones of genes. Their numbers and distribution patterns are an evolutionary mark for a gene family. We, therefore, compared the intron-exon structure of each *JcDof* gene. The results revealed that the gene structure pattern was consistent with the phylogenetic analysis. Based on the exon-intron structures, the number of introns varied from one to three in *J. curcas* (Figure 3b). There are ten *JcDof* genes with one intron (41.7%), 12 *JcDof* genes with two introns (50%), and two *JcDof* genes with three introns (8.3%). All of the *JcDof* genes in subfamily A possessed two introns, while the number of introns of the *JcDof* gene in subfamily C varied from one to three.

Our classification of *Dof* genes was also verified by the conserved motif analysis. All of the Dof protein sequences were loaded into the MEME analysis tool to identify the conserved motifs. As a result, a total of ten conserved motifs were observed, which were statistically-significant with *E*-values less than 1× 10^−40^ (Figure 3a, described in detail in Supplementary Figure S1 and Table S4). The motifs of Dof proteins identified by MEME were between 13–43 amino acids in length. Among them, Motif-1 is a common motif in all Dof proteins, corresponding to the CX2CX21CX2C single zinc-finger structure in the Dof domain, which was the highly-homologous core region of *Dof* family (Figure 3c). While all of the Group B proteins and many of the Group C1 and C2 proteins only contain Motif-1, some Dof proteins have extra specific motifs, which may be relevant to different functions. The Dof proteins from Group A had the most complicated motif patterns, and Motif-2, Motif-4, Motif-5, and Motif-9 were specific for them. While Group C members have relatively simple motif patterns compared with Group A, they also had group-specific motifs, such as Motif-6, Motif-8, and Motif-10, but not all the group members have these specific motifs. For further elucidation of the potential roles of the Group A specific motifs, we checked the GO annotations of the Group A genes in *Arabidopsis*. Interestingly, we found that comparing with the *Arabidopsis Dof* genes in other groups, most of the genes in Group A (5 out of 7) have some flower-development-related annotations, such as “flower development”, “negative regulation of long-day photo periodism”, “flowering”, “negative regulation of short-day photo periodism”, “regulation of timing of transition from vegetative to reproductive phase”, and “vegetative to reproductive phase transition of meristem”, which implied the possible function divergence of the *Dof* genes in group A (see Supplementary Table S3 for detailed information).

### 2.5. Chromosomal Locations and Gene Duplication Events of JcDof Genes

**Figure 4:**
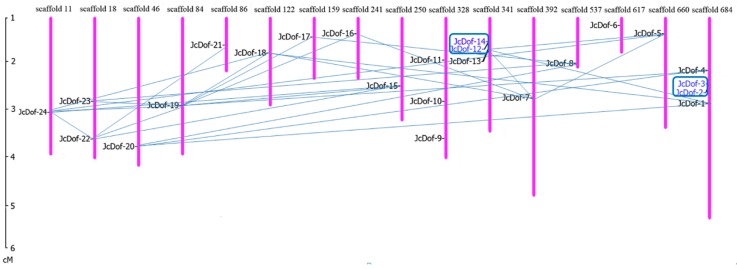
Chromosomal locations and gene duplication events of *JcDof*. Respective scaffold numbers are indicated at the top of each bar. The scale on the left is in centimorgan (cM). The *JcDof* gene pairs of segmental and tandem duplication are linked by pale blue lines and marked in dark blue rectangles, respectively. The detailed information of duplication pairs are described in Supplementary Table S5.

In order to explore the mechanism of evolution and amplification of *JcDof* gene, the chromosomal locations and gene duplication events of *JcDof* genes were further analyzed. The chromosomal distribution of *JcDof* genes was plotted using Map Inspect software (Figure 4). The duplication events of *JcDof* genes were also examined, and *Dof* gene-pairs arising from segmental and tandem duplication were marked with light blue line and dark blue rectangles, respectively. From Figure 4 we can find that some *Dof* genes, such as *JcDof-19*, have been duplicated several times to form more than one duplicated gene-pair with other genes; and some *JcDof* genes, such as *JcDof-15*, *JcDof-22*, and *JcDof-24*, are evolutionarily too close to resolve their gene duplication order (the duplication pairs are described in detail in Supplementary Table S5). The gene expansion of the *Dof* family in physic nut mainly resulted from segmental duplication, and tandem duplication also played a minor role. In total, 26 pairs of segmental duplicated *JcDof* genes (93% of all duplicated genes) and two pairs of tandem duplicated *JcDof* genes (7% of all duplicated genes) were found. For most of the duplicated gene pairs (22 out of 28), the pairwise *JcDof* genes often came from the same phylogenetic group, with very high sequence similarities. Specifically, tandem duplicated genes have higher sequence similarity than segmental duplicated genes (Table S5).

**Figure 5:**
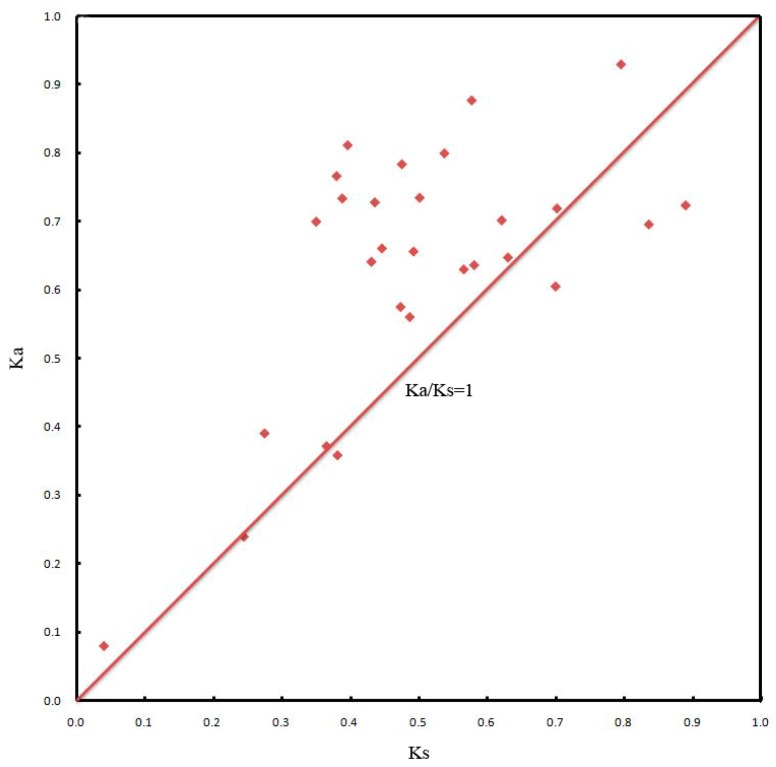
The *Ka*/*Ks* value of duplicated *JcDof* gene pairs. 28 pairs of duplicated *JcDof* genes and 23 pairs duplicated genes with *Ka*/*Ks* more than one. The detailed *Ka*/*Ks* information of duplication pairs are described in Supplementary Table S5.

To further understand the evolutionary constraints acting on all of the duplicated *JcDof* genes, we calculated the non-synonymous substitution rate (*Ka*), synonymous substitution rate (*Ks*) and *Ka*/*Ks* for all of the 28 pairs duplicated genes (Figure 5 and Table S5). We found 23 pairs duplicated genes whose *Ka*/*Ks* were more than one (accounting for 82% of all the duplicated genes) and five pairs duplicated genes whose *Ka*/*Ks* ratio were less than one (accounting for 18% of all the duplicated gene pairs) (Table S5). This implied that most of the *Dof* duplicated gene pairs tended to be subjected to positive selection, which may play important roles in the origin of adaptive phenotypes and the possible function divergence in *JcDof* genes.

### 2.6. Expression Patterns of JcDof Genesunder Different Abiotic Stress and Hormone Treatments

**Figure 6:**
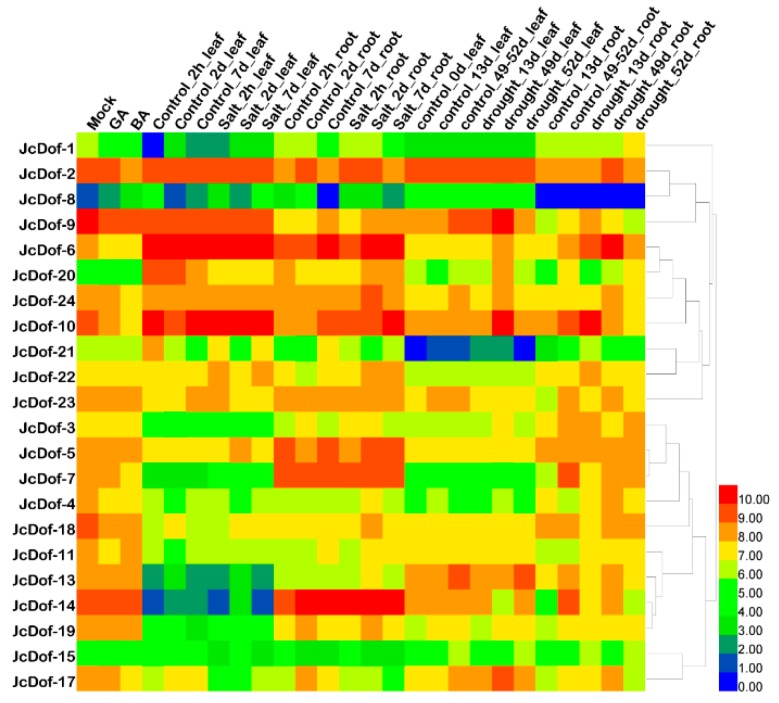
Expression patterns of *JcDof* genes under different treatments. The heatmap was generated by HemI software using the expression data of the *JcDof* genes, and normalized log_2_ transformed values were used with hierarchical clustering represented by the color scale (0–10). Blue indicates low expression, and red indicates high expression. The samples were: roots and leaves (salt- and drought-stressed at different times), and roots (BA treatment). The detailed information of expression data are described in Supplementary Tables S6 and S7.

In order to further study the possible function divergence of *JcDof* genes, we investigated the expression level of *JcDof* genes under various abiotic stresses and hormonal treatments by using the public transcriptome data from NCBI SRA database (Supplementary Tables S6 and S7 for detailed information). We employed a heatmap to visualize a global transcription profile of the *JcDof* genes. As shown in Figure 6, *JcDof* genes showed diverse responses to various treatments, and significant differences were found in response to 6-Benzylaminopurine (BA), salt, and drought treatments (two-fold increases or decreases compared to controls).

In the BA treatment experiments (gene expression data collected from roots), compared with the negative control (mock), three genes (*JcDof-1*, *JcDof-8*, and *JcDof-10*) exhibited significant responses. Among them, *JcDof-1* and *JcDof-10* showed reduced expression when responding to BA treatment, with more than two-fold (*JcDof-1*) and nearly four-fold (*JcDof-10*) decreasing, respectively. Meanwhile, *JcDof-8* showed a significantly up-regulated expression with more than four-fold increase. We further checked the GO annotations of their Arabidopsis orthologs, and found they were annotated as “seed coat development” (*JcDof-1*, *AT1G29160.1*), “guard cell differentiation, positive regulation of transcription, regulation of cell wall pectin metabolic process, stomatal movement” (*JcDof-8*, *AT5G65590.1*), and “flower development” (*JcDof-10*, *AT5G39660.1*) respectively, which may imply the possible roles of these three genes (Supplementary Table S3 for detailed information).

We further analyzed the expression patterns of the *JcDof* genes in salt- and drought-stressed roots and leaves at different times: 2 h, 2 days, and 7 days (salt-stressed); 13 days, 49 days, and 52 days (drought-stressed). The fold changes of gene expression were calculated between abiotic stress treatments and controls. Many *JcDof* genes exhibited significant responses, and some of them showed significant up- or down-regulation in both roots and leaves, such as *JcDof-8*, *JcDof-17*, and *JcDof-20* in salt-stressed treatments, and *JcDof-6*, *JcDof-8*, *JcDof-10*, *JcDof-14*, *JcDof-17*, and *JcDof-21* in drought-stressed treatments. Most of these significantly up- or down-regulated genes (seven out of nine) tended to show similar expression changes (up- or down-regulation) in both roots and leaves. The only two exceptions were *JcDof-20* and *JcDof-14*. *JcDof-20* showed significantly reduced expression in leaves (from 2 h to 7 days) when responding to salt treatment, while *JcDof-20* expression in salt-treated roots first decreased (at 2 h), and then increased significantly (two days and seven days). Another gene, *JcDof-14*, showed significantly reduced expression in leaves (in 49 days) when responding to drought treatment, while *JcDof-14* expression in drought-treated roots first increased (in 13 days), and then decreased significantly (49 days and 52 days).

We have also checked the differential expression patterns of the duplicated *JcDof* gene pairs, and found that if *JcDof* genes differentially expressed in some stress treatments, and their duplicated counterparts were more likely not to show differential expression (27 pairs vs. 20 pairs, Supplementary Table S8 for detailed information). We think these results are consistent with our *Ka*/*Ks* results, that most of the duplicated *JcDof* genes tended to be subjected to positive selection, and implied the possible function divergence in *JcDof* genes.

## 3. Discussion

The Euphorbiaceae family includes some of the most efficient biomass accumulators, such as physic nut, castor bean, cassava, and rubber tree [9,31]. Crop improvement in Euphorbiaceae for sustainable industrial raw materials and food production requires more extensive genome-wide studies on these species. Notably, physic nut has become an ideal model organism in Euphorbiaceae for further functional genomics analysis due to its sequenced genome, genetic linkage map, and abundance of high-throughput transcriptome data. Studies on physic nut will provide insights into the investigation of other Euphorbiaceae organisms.

Genome-wide gene family analysis is abasic and a key step to understanding the gene structure, function, and evolution [32]. The *Dof* gene family has been shown to play crucial roles in the regulatory network of plant defense, including responses to diverse biotic and abiotic stresses [22,23,33,34]. Until now, the *Dof* genes have been identified and characterized in different plant species, but not in the promising energy plant physic nut yet. Therefore, we conducted a comprehensive analysis of the *JcDof* family in physic nut, along with their homologs in *R. communis* and *A. thaliana*, to study their phylogenetic relationships and potential functions.

In total, we identified 24 *JcDof* genes in the physic nut genome. Compared with the number of *Dof* genes in *A. thaliana* (36 genes from TAIR), the size of physic nut *Dof* gene family is much smaller [35], although the assembled genome size of physic nut is approximately three times larger than the *A. thaliana* genome (320.5 Mbp vs. 125 Mbp) [9,36]. Correspondingly, we had discovered that the members from Group B, one of the major groups in the phylogenetic tree, all pertained to *AtDof* genes. In addition, Subgroup C1 contained 13 *AtDof* genes; while only nine *JcDof* genes were noted. Subgroup C2 had 12 *AtDof* genes and 10 *JcDof* genes. These results suggested that *JcDof* and *AtDof* genes should arise through different duplication events, and might have undergone species/lineage-specific gene gain or loss. 

Both tandem duplication and segmental duplication contributed to the variation in gene family number and distribution [37,38]. In total, 26 gene-pairs from segmental duplication and two from tandem duplication were found in physic nut. We calculated the *Ka*/*Ks* ratios for these duplicated *JcDof* paralog genes, and found most of the duplicated genes pairs had *Ka*/*Ks* ratios over 1, implying that positive selection played an important role in the evolution of *JcDof* genes, and high-throughput expression data analysis further confirmed the functional diversity of *JcDof* genes. *JcDof* genes showed diverse responses to various treatments, and might participate in different stress/hormone-responding regulatory processes. This work provides valuable information for understanding the evolution of *JcDof* genes and lays a foundation for future functional analysis of *Dof* genes in the process of growth, development, and Dof-mediated regulation in physic nut.

## 4. Materials and Methods

### 4.1. Data Sources

The physic nut genomic and proteomic sequences were downloaded from the NCBI database (Available online: https://www.ncbi.nlm.nih.gov/, Assembly JatCur_1.0). The Dof protein sequences of *A. thaliana* were obtained from the Arabidopsis genome database (TAIR 9.0 release, Available online: http://www.arabidopsis.org/) [35]. The Dof protein sequences of castor bean were obtained from the PlantTFDB database (Available online: http://planttfdb.cbi.pku.edu.cn/) [16]. The physic nut gene expression data were collected from the SRA database (Available online: https://www.ncbi.nlm.nih.gov/) [39].

### 4.2. Dof Gene Identification and Characterization

To identify all the possible *Dof* genes in physic nut, both local BLASTP [40] and Hidden Markov model (HMM) searches were performed [41]. For BLASTP, the known Dof proteins from Arabidposis were taken as queries and the *E*-value was set to 1 × 10^−10^. For the HMM search, the HMM profile of the Dof domain was used as query and the *E*-value was set to 1 [24]. All the retrieved sequences were further scanned and tested using SMART (Available online: http://smart.embl-heidelberg.de/) [42] and NCBI Conserved Domains database (Available online: http://www.ncbi.nih.gov/Structure/cdd/cdd.shtml) for authentication of the presence of Dof domain [43]. We manually removed redundant sequences that do not have Dof domain or have incomplete encoding frame. Parameters, such as protein length, molecular weight, isoelectric point, and instability coefficient of all the Dof proteins in physic nut were predicted using ExPASy Proteomics Server (Available online: http://prosite.expasy.org/) [44]. The orthologous genes of JcDof proteins in *A. thaliana* were predicted by BLASTP.

### 4.3. DNA-Binding Domain Conservation Analysis of JcDof Protein

The conserved regions of JcDof proteins were extracted by DNAMAN tool (version 2.6 Lynnon Biosoft, Quebec City, QC, Canada) [45]. We then identified highly-conserved Dof domain for all Dof proteins by multiple sequence alignment analysis using ClustalW MEGA integration software [46].

### 4.4. Phylogenetic Analysis

Physic nut, *A. thaliana*, and *R. communis* Dof protein sequences were pretreated by GUIDANCE2 online tool to remove unreliable columns [47]. The phylogenetic relationship among the Dof proteins was analyzed using ClustalW and the dendrogram was constructed using MEGA (v6.0, Tokyo Metropolitan University, Tokyo, Japan) by neighbor-joining method, with the following parameters: Poisson correction, pairwise deletion, and 1000-bootstrap replicates [48].

### 4.5. Gene Structure of Dof Proteins

Positional information for both the gene sequences and the corresponding coding sequences was loaded into the Gene Structure Display Server (GSDS v2.0, Available online: http://gsds.cbi.pku.edu.cn/) to obtain information on intron/CDS structure [49]. The coordinates of the Dof domain in each protein were recalculated into the coordinates in the corresponding gene sequence and featured in the gene structure.

### 4.6. Detection of Additional Conserved Motifs

To identify additional conserved motifs outside the Dof domain of physic nut Dof proteins, we used Multipel Expectation Maximization for Motif Elucidation (MEME v4.11.2, Available online: http://meme.nbcr.net/meme/) [50]. The limits on maximum width, minimum width, and maximum number of motifs were specified as 5, 150, and 10, respectively. The motifs were numbered serially according to their order in MEME. Those motifs common to genes in one of the three similarity groups were designated as the group-specific signatures.

### 4.7. Chromosomal Localization

According to the chromosomal positions of genes, we drew a map of the distribution of *Dof* genes throughout the physic nut genome using MapInspect software (Available online: http://mapinspect.software.informer.com/) [51]. The *Dof* gene pairs resulting from segmental or tandem duplication were linked by lines and marked in blue rectangle, respectively.

### 4.8. Detection of Gene Duplication Events and Estimation of Synonymous (Ks) and Nonsynonymous (Ka) Substitutions per Site and Their Ratio

Duplicated gene pairs derived from segmental or tandem duplication were identified in physic nut genome based on the method described in the Plant Genome Duplication Database [52,53]. An all-against-all BLASTP comparison (*E*-value ≤ 1 × 10^−20^) provided the gene pairs for syntenic clustering determined by MCScanX (*E*-value ≤ 1 × 10^−20^) [54]. Tandem duplication arrays were identified using BLASTP with a threshold of *E*-value < 1× 10^−20^, and one unrelated gene among cluster members was tolerated, as described for *A. thaliana*. Pairs from segmental and tandem duplications were used to estimate *Ka*, *Ks*, and their ratio. Coding sequences from segmentally and tandemly duplicated *Dof* gene pairs were aligned by PRANK [55] and trimmed by Gblocks. The software DnaSP (Available online: http://www.softpedia.com/get/Science-CAD/DnaSP.shtml) [56] was then used to compute *Ka* and *Ks* values for each pair following the YN model (a simple model of voting) [57]. If *Ka*/*Ks* > 1, there is positive selection pressure; if *Ka*/*Ks* = 1, there is neutral selection or natural selection pressure; if *Ka*/*Ks* < 1, there is a purification selection effect [58,59].

### 4.9. Expression Analysis of Physic Nut Dof Genes

The original expression data for *JcDof* genes under different treatments (including gibberellins [GA], 6-Benzylaminopurine[BA], high salt concentration and drought) were retrieved from NCBI SRA database (Available online: https://www.ncbi.nlm.nih.gov/). All the data were analyzed using Tuxedo suite (TopHat and Cufflinks, http://post.queensu.ca/~rc91/NGS/TuxedoTutorial.html) and then upper-quartile normalized and log transformed. Heat maps were generated by means of the HemI toolkit (Available online: http://hemi.biocuckoo.org/) with average linkage hierarchical clustering [60,61].

## 5. Conclusions

In conclusion, a total of 24 *Dof* genes were identified from physic nut, and these *Dof* genes were further divided into three major groups based on the phylogenetic inference. The gene structures, conserved motifs, gene duplicated events, selection pressures, and expression profiling of these *JcDof* genes were analyzed. A genome comparison discovered that the expansion of the *Dof* gene family in physic nut mainly resulted from segmental duplication, and this expansion was mainly subjected to positive selection. The expression profile demonstrated the broad involvement of *JcDof* genes in different hormonalor abiotic stressed treatments. Among them, three genes (*JcDof-1*, *JcDof-8*, and *JcDof-10*) exhibited significant responses to the BA treatment. Furthermore, many *JcDof* genes were significantly responsive to the salt and drought treatments. On the whole, this study provides an extensive resource for understanding the *Dof* genes in physic nut.

## Figures and Tables

**Table 1:** The information of the *JcDof* gene family.

Gene ID	Protein Name	Protein Model	Position of Dof Domain	Protein Length	Mw	pI	Instability Index	Orthologous
105649666	JcDof-1	XP_012091770.1	40–98	160	18,217	9.4	49.94	AT1G29160.1
105649561 *	JcDof-2.1	XP_012091631.1	67–125	331	36,503	8.74	58.25	AT1G28310.2
	JcDof-2.2	XP_012091630.1	67–125	365	40,036	8.72	60.68	AT1G28310.2
105649560 *	JcDof-3.1	XP_012091629.1	39–97	321	34,862	9.42	61.1	AT5G60850.1
	JcDof-3.2	XP_012091628.1	39–97	321	34,862	9.42	61.1	AT5G60850.1
105649506	JcDof-4	XP_012091561.1	48–106	283	31,234	8.87	58.45	AT1G28310.2
105645621	JcDof-5	XP_012086658.1	39–97	302	32,787	8.69	56.53	AT4G24060.1
105644078	JcDof-6	XP_012084716.1	148–206	518	55,717	5.88	43.82	AT5G62430.1
105642820 *	JcDof-7.1	XP_012083166.1	38–96	256	27,889	9.04	58.51	AT3G61850.4
	JcDof-7.2	XP_012083165.1	41–99	259	28,222	9.04	57.94	AT3G61850.4
	JcDof-7.3	XP_012083164.1	23–81	272	29,648	8.89	61.74	AT3G61850.4
	JcDof-7.4	XP_012083162.1	23–81	272	29,648	8.89	61.74	AT3G61850.4
	JcDof-7.5	XP_012083161.1	23–81	272	29,648	8.89	61.74	AT3G61850.4
	JcDof-7.6	XP_012083160.1	38–96	287	31,275	8.87	59.86	AT3G61850.4
	JcDof-7.7	XP_012083159.1	41–99	290	31,607	8.87	59.35	AT3G61850.4
105641716	JcDof-8	XP_012081705.1	32–90	344	36,852	8.97	52.37	AT5G65590.1
105640671	JcDof-9	XP_012080436.1	31–89	334	36,528	6.86	57.05	AT5G60850.1
105640546	JcDof-10	XP_012080278.1	121–179	471	51,491	6.61	54.99	AT5G39660.1
105640379	JcDof-11	XP_012080063.1	52–110	312	34,986	6.75	39.5	AT5G62940.1
105639962	JcDof-12	XP_012079559.1	26–84	236	24,419	9.17	48.6	AT3G50410.1
105639655	JcDof-13	XP_012079164.1	63–121	326	34,900	9.08	48.8	AT1G07640.3
105639642	JcDof-14	XP_012079148.1	26–84	246	25,139	8.72	41.9	AT3G50410.1
105636282 *	JcDof-15.1	XP_012074917.1	75–133	353	36,578	9.14	50.3	AT2G37590.1
	JcDof-15.2	XP_012074916.1	84–142	362	37,605	9.14	50.73	AT2G37590.1
105635894	JcDof-16	XP_012074418.1	10–68	282	30,892	5.14	48.76	AT3G52440.1
105633699	JcDof-17	XP_012071724.1	21–79	245	25,883	8.52	39.4	AT1G47655.1
105632564	JcDof-18	XP_012070363.1	101–159	497	53,629	7.78	43.27	AT3G47500.1
105631489	JcDof-19	XP_012069011.1	18–76	249	26,497	8.26	47.16	AT3G21270.1
105630455	JcDof-20	XP_012067660.1	129–187	465	51,091	6.8	47.74	AT5G39660.1
105629142	JcDof-21	XP_012066060.1	28–86	287	32,762	4.65	51.26	AT1G21340.1
105628246	JcDof-22	XP_012065018.1	71–129	315	33,921	9.23	51.88	AT2G28810.1
105628152	JcDof-23	XP_012064896.1	36–94	290	32,430	6.65	41.41	AT2G28510.1
105647749	JcDof-24	XP_012089351.1	70–128	338	35,691	9.19	50.23	AT3G55370.3

* These genes are regulated by alternative splicing mechanisms. Mw: Molecular weight; pI: Isoelectric point.

## Supplementary Materials

The following are available online at http://www.mdpi.com/1422-0067/19/6/1598/s1.

## Abbreviations

TF	Transcription factor
HMM	Hidden Markov model
Mw	Molecular weight
pI	Isoelectric point
GA	Gibberellic acid
BA	6-Benzylaminopurine
