# Supplementary figures and images for: Genome-Wide Screening and Characterization of the Dof Gene Family in Physic Nut (Jatropha curcas L.)
Source: Int J Mol Sci. 2018 May 29;19(6):1598. doi: 10.3390/ijms19061598 (PMC6032415; doi:10.3390/ijms19061598)

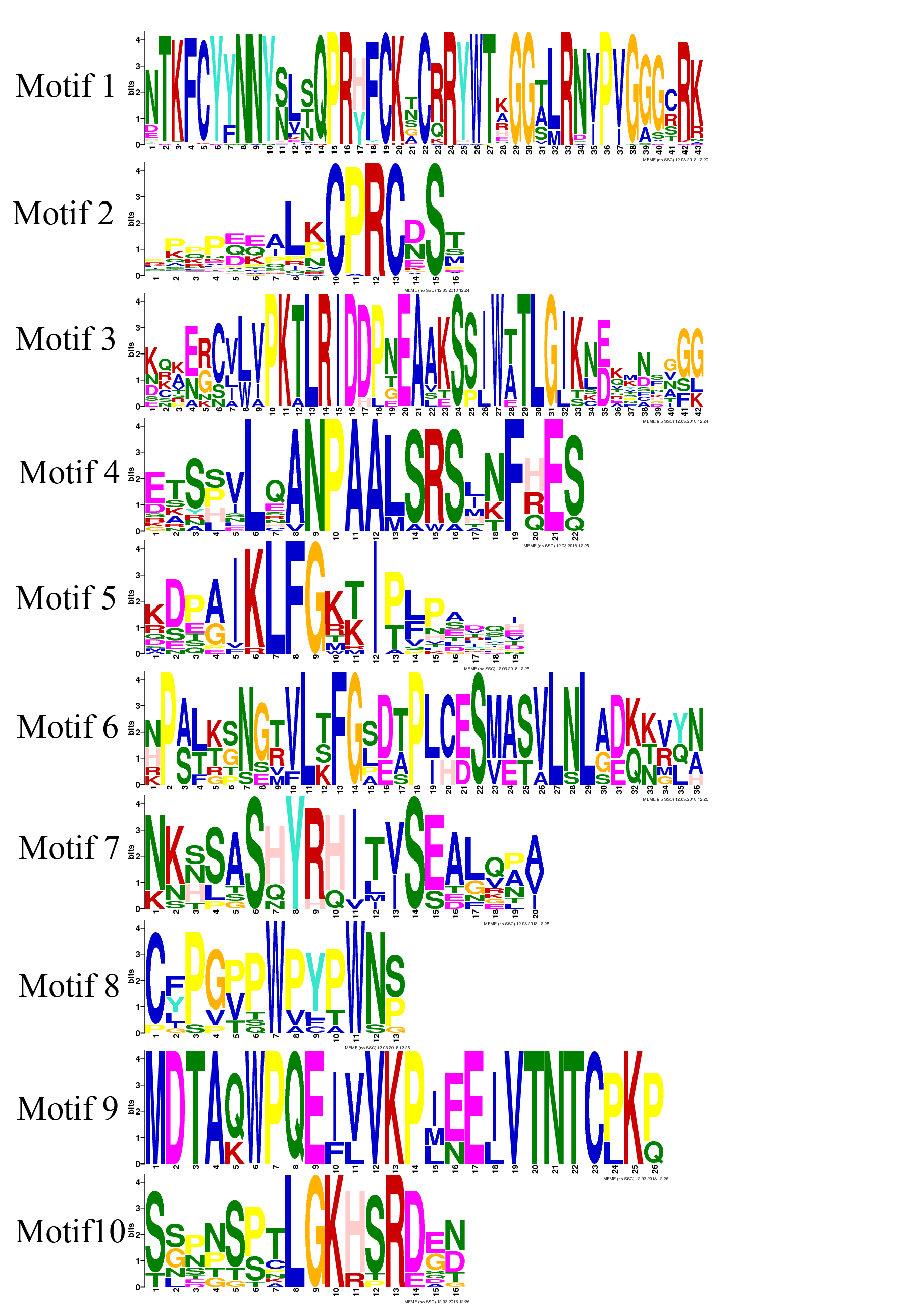

Supplement: Supplementary file 1 [file ijms-19-01598-s001.zip › Supplementary Tables and Figures/Figure S1. Sequence logos of the motifs in Dof proteins.tif]
